# Urinary Virome Perturbations in Kidney Transplantation

**DOI:** 10.3389/fmed.2018.00072

**Published:** 2018-04-19

**Authors:** Tara K. Sigdel, Neil Mercer, Sharvin Nandoe, Carrie D. Nicora, Kristin Burnum-Johnson, Wei-Jun Qian, Minnie M. Sarwal

**Affiliations:** ^1^Department of Surgery, University of California, San Francisco, San Francisco, CA, United States; ^2^Erasmus MC, University Medical Center, Rotterdam, Netherlands; ^3^Biological Sciences Division, Pacific Northwest National Laboratory, Richland, WA, United States

**Keywords:** kidney transplantation, virome, urine, proteomics, biomarkers

## Abstract

The human microbiome is important for health and plays a role in essential metabolic functions and protection from certain pathogens. Conversely, dysbiosis of the microbiome is seen in the context of various diseases. Recent studies have highlighted that a complex microbial community containing hundreds of bacteria colonizes the healthy urinary tract, but little is known about the human urinary viruses in health and disease. To evaluate the human urinary virome in the context of kidney transplantation (tx), variations in the composition of the urinary virome were evaluated in urine samples from normal healthy volunteers as well as patients with kidney disease after they had undergone kidney tx. Liquid chromatography-mass spectrometry/mass spectrometry analysis was undertaken on a selected cohort of 142 kidney tx patients and normal healthy controls, from a larger biobank of 770 kidney biopsy matched urine samples. In addition to analysis of normal healthy control urine, the cohort of kidney tx patients had biopsy confirmed phenotype classification, coincident with the urine sample analyzed, of stable grafts (STA), acute rejection, BK virus nephritis, and chronic allograft nephropathy. We identified 37 unique viruses, 29 of which are being identified for the first time in human urine samples. The composition of the human urinary virome differs in health and kidney injury, and the distribution of viral proteins in the urinary tract may be further impacted by IS exposure, diet and environmental, dietary, or cutaneous exposure to various insecticides and pesticides.

## Introduction

The human microbiome has been studied extensively in various biofluids, such as blood (1), urine (2–4), saliva (5, 6), cerebrospinal fluid (7), and bronchoalveolar lavage (8, 9), for its influence on human health and disease (10). Several reports on the assessment of microbiomes in different regions of human body have been reported including lung and gut (11–15). However, only little is known about the urinary microbiome and its changes in the context of kidney injury after kidney transplantation (tx) (16, 17). Most human microbiome studies have mapped 16S rRNA for bacterial profiling (18), and there is only a small body of data examining the urine virome (19, 20), and a single published study has used next-generation sequencing to map viral genomic components in the urine of kidney transplant patients (19).

Dysbiosis of the microbiome is associated with multiple diseases such as inflammatory bowel disease, colon cancer, obesity and pulmonary disease (21–25), but little is known about alterations of the virome in human body fluids, such as saliva, bronchoalverolar lavage and urine. It is recognized that various components of the microbiome perform essential functions including biosynthesis of cofactors and vitamins, metabolism of essential compounds, and barrier protection from pathogens (26–28). Similar dysbiotic and protective functions may also be ascribed to the urine virome; hence, it is important to ascertain the composition of the human virome in health and disease. For purposes of this study, we chose to conduct these studies in urine in health and in the context of IS exposure after kidney tx, during stable kidney function and in the context of kidney tx injury. The focus of the study was to evaluate the repertoire of viruses as determined by the peptides present in human urine and their perturbations after IS exposure and various causes of acute, chronic, and infectious kidney injury.

The microbiome changes over time and correlates with organism diversity. Microbiome analysis can be segregated into both structural analysis based on operational taxonomic units based on sequence phylogeny, and functional analysis based on metagenomics sequencing and proteomics (29, 30). Identified microbiota in different diseases can be further studied by cultivation, functional metagenomics, and multiplexed immunofluorescence and *in situ* hybridization. The NIH human microbiome project has published the human microbiome in 15 body sites from 300 individuals (31).

## Materials and Methods

**Figure 1:**
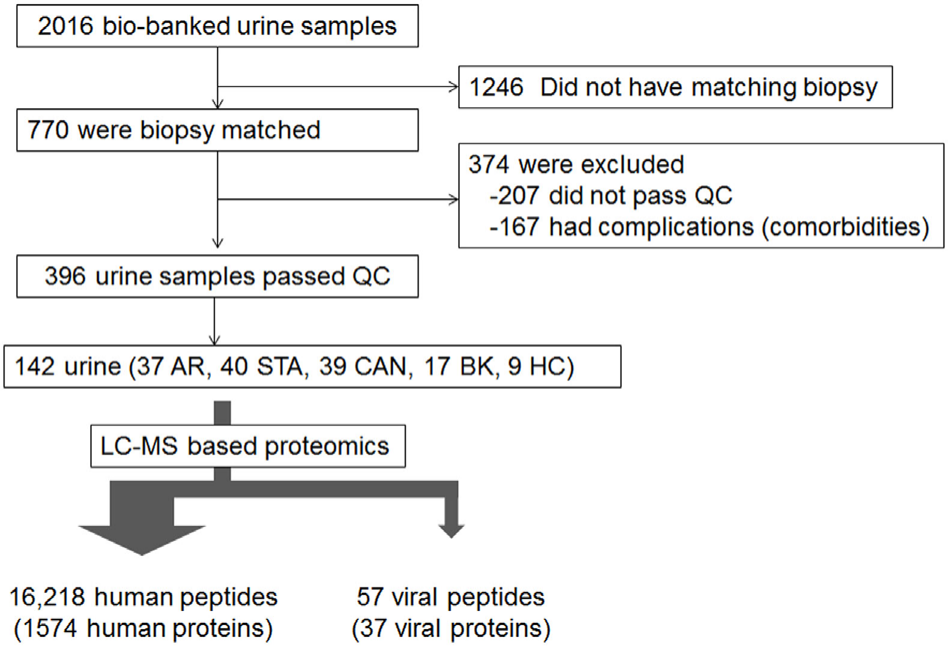
Source of samples. LC-MS based proteomics was performed on the 142 samples chosen: 37 with acute rejection (AR), 40 stable (STA), 39 with chronic allograft nephropathy (CAN), 17 with BK virus nephritis, and 9 healthy controls.

A total of 142 unique samples were evaluated from a biorepository containing 2016 collected by IRB approved informed consent from adult and pediatric samples from the kidney tx programs at Stanford University and University of California San Francisco, between urine samples of which 770 were accompanied with matched kidney tx bx with centralized pathology histology reads and compartment scores using the standardized Banff schema (32) for scoring kidney tx bx injury (Figure 1). The study was approved by The Human Research Protection Program of the University of California, San Francisco. The urine samples were phenotyped based on the matched kidney bx pathology into five groups: healthy control (HC; *n* = 9), Stable graft (STA; *n* = 40), acute rejection (AR; *n* = 37), chronic allograft nephropathy (CAN; *n* = 39), and BK virus nephritis (BKVN; *n* = 17). Urine was centrifuged 2,000 × *g* at 4°C for 20 min to get rid of urine sediments. The supernatant was passed through a filer membrane of 10 kDa to remove native peptides from intact proteins larger than 10 kDa in size. The total protein was then trypsin digested and the resulting tryptic peptides were analyzed by LC-MS platform (Orbitrap Velos MS). The detail methods of protein preparation and analyses are reported elsewhere (33).

The MSGF plus customized algorithm generated by our group (https://omics.pnl.gov/software/ms-gf), was used to search MS/MS spectra against the combined human protein sequence database and the NCBI viral database. Peptides were initially identified from database searching applying the following criteria: MSGF spectrum E-value (a probability value of the peptide to MS/MS spectrum match with the lower value the higher probability to be correct match) to be <10-10, Peptide level Q-value (false discovery rate estimated by targeted-decoy database search) to be <0.01, and mass measurement error <10 ppm (±5 ppm). The decoy database searching methodology was used to confirm the final false discovery rate at the unique peptide level to be <1%. Due to the anticipated higher false discovery rate for peptides from viral proteins, a more stringent filtering criteria with MSGF spectrum E value to be <1E-13 was applied. The false discovery rate was estimated to nearly 0% based on the well-accepted target-decoy searching strategy because no decoy hits were observed following this stringent cutoff. Data are shown as percentages and mean ± SD. Comparisons of different categories are done using ANOVA and *p* values of <0.05 are considered significant.

## Results

Our group has previously published a detailed analysis of biologically relevant human proteins in these urine samples collected from kidney transplant recipients with different graft injury phenotypes, as confirmed by matched kidney transplant histopathology on the biopsy, collected at the same time as the urine sample; this data has been deposited in the proteomic MassIVE repository (accession MSV000079262) and in the ProteomeXchange repository (accession PXD002761) (33). In this study, we only focused on the identification and analysis of viral proteins in the same cohort of kidney transplant patients, with the inclusion also of age- and gender-matched healthy control human urine samples to evaluate viral proteins in both health and kidney injury. It is important to note that of the total analyzed human and viral proteins in urine, viral proteins alone constitute <0.2% of the total identified proteins, highlighting the very low abundance of rather rare viral proteins in human urine, irrespective of the type of kidney injury, and irrespective of baseline immunosuppression usage.

**Figure 2:**
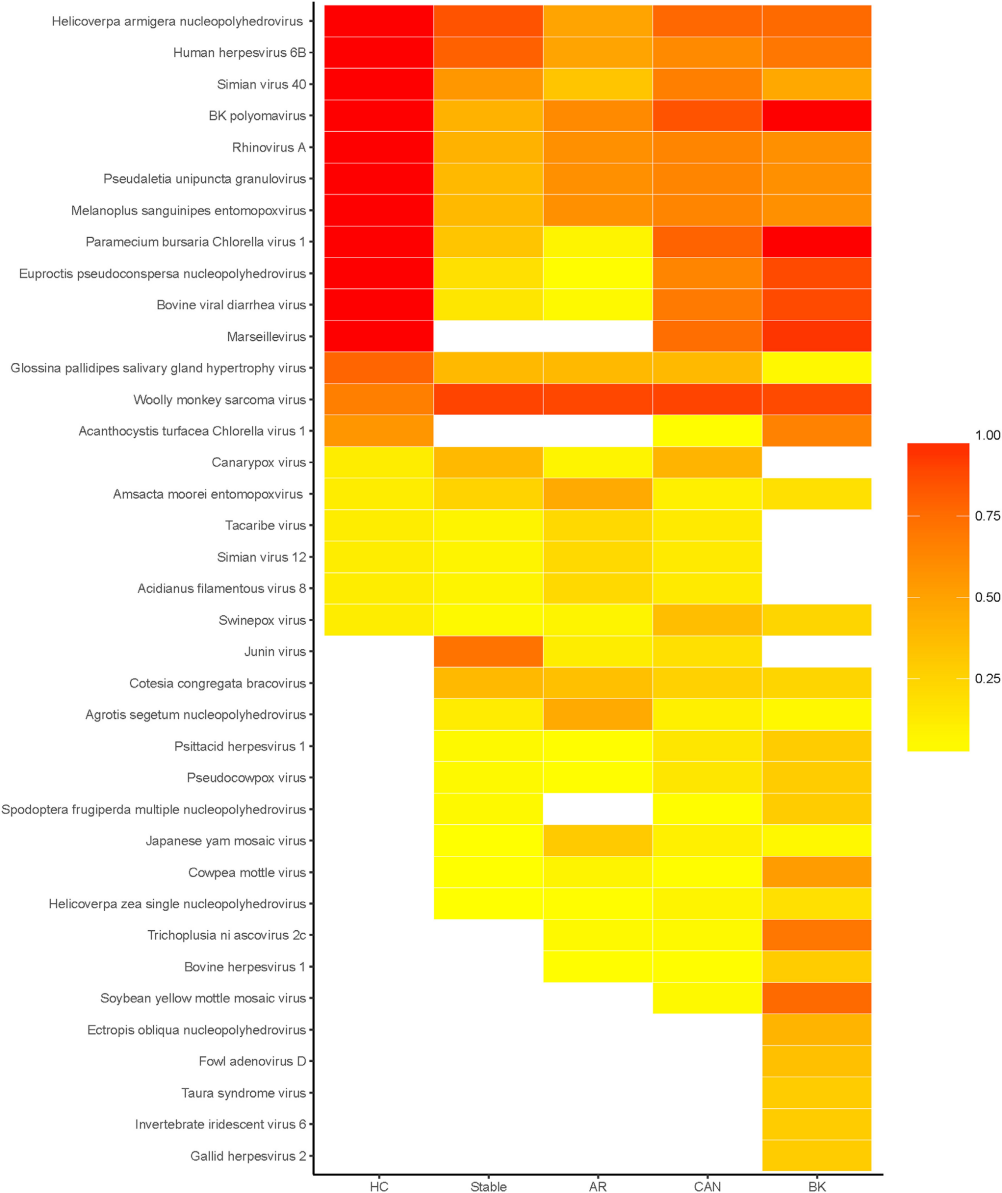
Heatmap of prevalence by disease status. Heatmap of the percentage of patients, within each disease status, in which each virus was detected. Total number of unique viruses in each disease status: healthy control (HC) 20, stable 27, acute rejection (AR) 28, chronic allograft nephropathy (CAN) 32, and BK 32.

The results presented in this paper come from viral peptide mapping, unlike genomic sequencing of viral components as reported by previous publications (19). As an initial analysis, we focus on the prevalence of urinary viral proteins specific to each kidney transplant phenotype of STA, AR, CAN, and BKVN, and the variations noted over the healthy control urine virome. Urinary viral protein data for each sample was evaluated for sample abundance relative to the mean level of that virus in the entire sampled population. We found that on an average, 22% (range 4–67%) of kidney transplant patients had different viral proteins detected in their urine, excluding patients with BKVN, where detection of the BK virus in urine is to be expected, as these patients have infection with BK virus in their urinary tract and in the kidney transplant. The distribution of different viral proteins in different tx injury states and normal health are shown in the prevalence heat map (Figure 2). Overall, a total of 57 viral proteins from 37 unique viruses were identified, many of these being unexpected and previously not described as “commensals” in human urine. Of these unique viruses, 8 viruses were single-stranded RNA viruses, 1 virus was a single-stranded RNA-RT virus, and 28 viruses were double-stranded DNA viruses (Table 1). Out of the 142 patients, all patients had at least one viral protein in their urine, with an average of 10.23 ± 4.57 viral proteins/sample.

**Table 1:** Breakdown of unique viruses.

Virus	Virus family	Virus type	Host	Described in humans	Used as pesticide/insecticide
*Acanthocystis turfacea* Chlorella virus 1	Phycodnaviridae	dsDNA	Algae	Yes	No
Acidianus filamentous virus 8	Lipothrixviridae	dsDNA	Sulfo bacteria	No	No
*Agrotis segetum* nucleopolyhedrovirus	Baculoviridae	dsDNA	Lepidoptera	No	No
*Amsacta moorei* entomopoxvirus	Poxviridae	dsDNA	Butterfly	No	No
BK polyomavirus	Polyomaviridae	dsDNA	Humans	Yes	No
Bovine herpesvirus 1	Herpesviridae	dsDNA	Cow	No	No
Bovine viral diarrhea virus	Flaviviridae	(+)ssRNA	Cow	No	No
Canarypox virus	Poxviridae	dsDNA	Canary	No	No
*Cotesia congregata* bracovirus	Polydnaviridae	dsDNA	Wasp	No	No
Cowpea mottle virus	Tombusviridae	(+)ssRNA	Cowpeas	No	No
*Ectropis obliqua* nucleopolyhedrovirus	Baculoviridae	dsDNA	Butterfly	No	Yes
*Euproctis pseudoconspersa* nucleopolyhedrovirus	Baculoviridae	dsDNA	Butterfly	No	Yes
Fowl adenovirus D	Adenoviridae	dsDNA	Birds	No	No
Gallid herpesvirus 2	Herpesviridae	dsDNA	Chicken	No	No
*Glossina pallidipes* salivary gland hypertrophy virus	Hytrosaviridae	dsDNA	Tsetse fly	No	No
Helicoverpa armigera nucleopolyhedrovirus	Baculoviridae	dsDNA	Butterfly	No	Yes
*Helicoverpa zea* single nucleopolyhedrovirus	Baculoviridae	dsDNA	Butterfly	No	Yes
Human herpesvirus 6B	Herpesviridae	dsDNA	Humans	Yes	No
Invertebrate iridescent virus 6	Iridoviridae	dsDNA	Insects	No	No
Japanese yam mosaic virus	Potyviridae	(+)ssRNA	Yam	No	No
Junin virus	Arenaviridae	(−)ssRNA	Mice	Yes	No
Marseillevirus	Marseilleviridae	dsDNA	Amoeba	Yes	No
*Melanoplus sanguinipes* entomopoxvirus	Poxviridae	dsDNA	Grasshoppers	No	No
Paramecium bursaria Chlorella virus 1	Phycodnaviridae	dsDNA	Algae	No	No
*Pseudaletia unipuncta* granulovirus	Baculoviridae	dsDNA	Moth	No	No
Pseudocowpox virus	Poxviridae	dsDNA	Cow	No	No
Psittacid herpesvirus 1	Herpesviridae	dsDNA	Parrots	No	No
Rhinovirus A	Picornaviridae	(+)ssRNA	Humans	Yes	No
Simian virus 12	Polyomaviridae	dsDNA	Baboons	No	No
Simian virus 40	Polyomaviridae	dsDNA	Humans	Yes	No
Soybean yellow mottle mosaic virus	Tombusviridae	(+)ssRNA	Soybean	No	No
*Spodoptera frugiperda* multiple nucleopolyhedrovirus	Baculoviridae	dsDNA	Lepidoptera	No	No
Swinepox virus	Poxviridae	dsDNA	Boars	No	No
Tacaribe virus	Arenaviridae	(−)ssRNA	Rodents	Yes	No
Taura syndrome virus	Dicistroviridae	(+)ssRNA	Shrimp	No	No
Trichoplusia ni ascovirus 2c	Ascoviridae	dsDNA	Moths	No	No
Wooly monkey sarcoma virus	Retroviridae	ssRNA-RT	Wooly Monkeys	No	No

### Prevalence of Human Urine Viral Proteins

**Figure 3:**
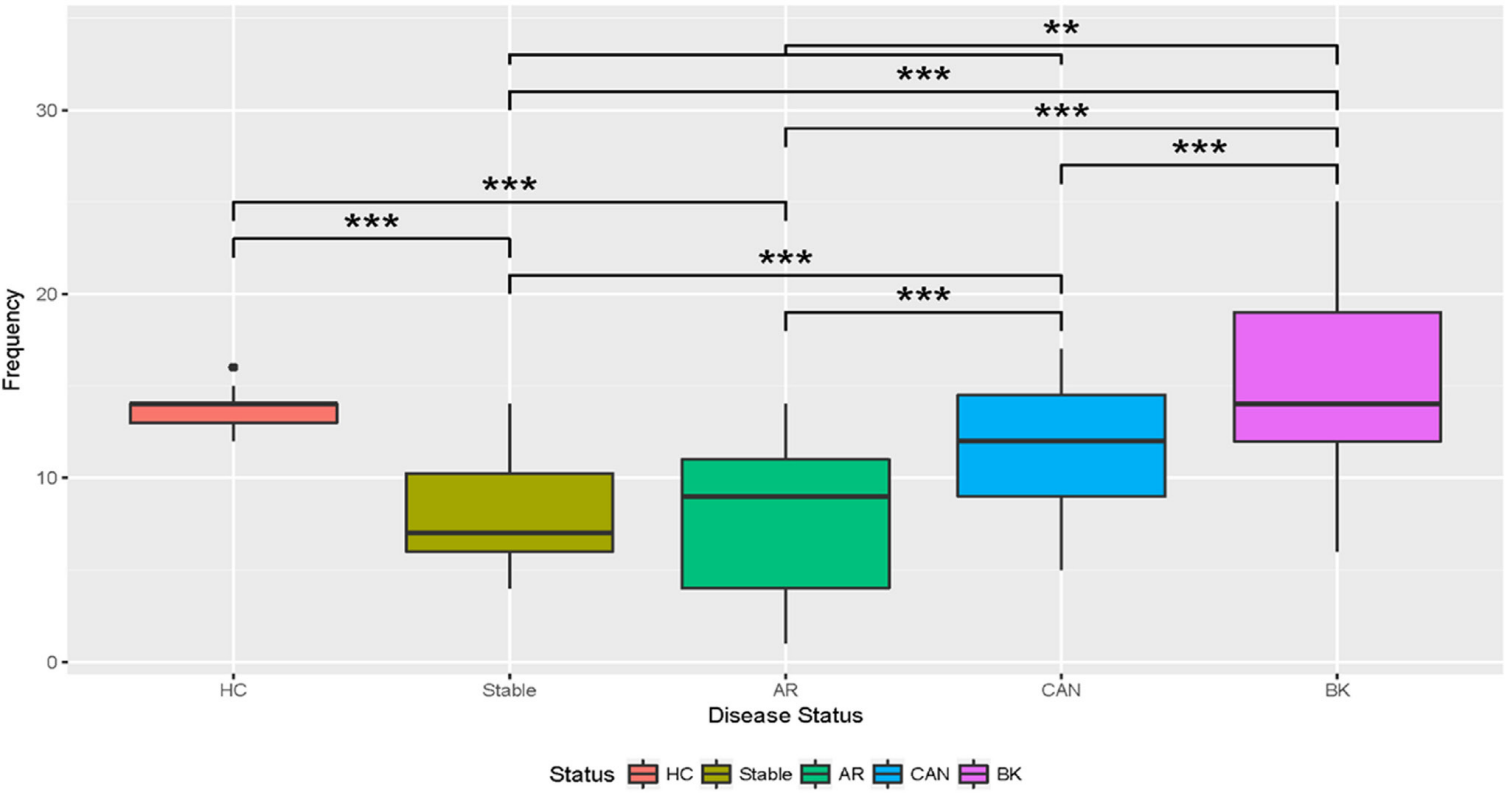
Boxplots of the number of unique viruses discovered by disease status. The boxplots show that the relative abundance of unique viruses discovered in stable and acute rejection (AR) patients is lower than the number discovered in healthy control (HC). The number of viruses discovered increased in chronic allograft nephropathy (CAN) above stable and AR. The number of viruses increased in BK over all immunosuppressed categories. *T*-Tests with Bonferroni corrections were used for multiple testing. *Significant at *p* < 0.05; **significant at *p* < 0.005; ***significant at *p* < 0.001.

The healthy control group examined had 20 unique viral proteins with a group average of 13.67 ± 1.32 viral proteins. Kidney tx patients with maintenance IS and stable kidney tx function had a statistically significant *lower* number of unique viruses in the group (8.08 ± 2.78; *p* = 7e-10) when compared to the healthy cohort (normal kidney function and a normal immune system), without any IS exposure. Nine new viral proteins are instead detected in the STA cohort compared to healthy controls, belonging to the following viruses: Junin virus, *Cotesia congregata* bracovirus, *Agrotis segetum* nucleopolyhedrovirus, Psittacid herpesvirus 1, Pseudocowpow virus, *Spodoptera frugiperda* multiple nucleopolyhedrovirus, Japanese yam mosaic virus, Cowpea mottle virus, and *Helicoverpa zea* single nucleopolyhedrovirus. Kidney injury after tx (despite continued IS exposure), results in an overall *increase* in the number of unique viral proteins over the STA tx cohort, with a significant increase in urine viral proteins in the CAN patient group (11.46 ± 3.52; *p* = 8e-10), and the BKVN patient group (15.76 ± 5.65; *p* = 13.7e-10). The repertoire of urine viral proteins appears to be quite distinct in different tx injury categories (Figure 3). The prevalence of BKV viral proteins in urine increases to 60–70% in AR, 70–80% in patients in CAN, and 100% in patients with BKVN (Figure 2), highlighting that increasing abundance of the BK virus in urine may result from augmentation of IS, as seen in the AR category, and with greater time post-tx, as seen in the CAN category. BKVN urine samples show the maximal divergence of urinary viral proteins, as expected. Four viral proteins that are consistently present in all other samples, inclusive of normal healthy controls (Canarypox virus, Tacaribe virus, Simian virus 12, Acidianus filamentous virus 8), are no longer observed in the BKVN cohort. As BKV is a DNA virus, we examined if the changes in the urinary virome are largely related to an emergence of new DNA viruses in BKVN. Five new viral proteins are noted only in the BKVN cohort (*Ectropis obliqua* nucleopolyhedrovirus, Fowl adenovirus D, Taura syndrome virus, Invertebrate iridescent virus 6, Gallid herpesvirus 2). 4/5 of BKVN cohort viruses were double-stranded DNA viruses (Table 1).

### Abundance of Human Urine Viral Proteins

We next evaluated the abundance of different viral proteins across all tx patients and HC urine samples. Among the 37 unique viruses examined, the following viruses were the most abundant in STA IS patients (Acidianus filamentous virus 8, *Agrotis segetum* nucleopolyhedrovirus, BK polyomavirus, Canarypox virus, *Cotesia congregata* bracovirus, Cowpea mottle virus, *Glossina pallidipes* salivary gland, hypertrophy virus, Simian virus 12, Tacaribe virus), and increased abundance of the Marsellavirus is seen in both CAN and BKVN. Despite intrarenal infection with the BKV virus in BKVN patients, we observe that low levels of the BK virus are detected also in all examined tx categories and healthy controls, likely as the BK virus is a normal human urine commensal, with its persistence in the human uroepithelium at low levels in almost all examined HC and kidney tx patients is not unexpected.

## Discussion

A study the role of the microbiome in organ transplantation (13, 17, 34) takes in to account of the impact of food intake, digestion, metabolism, and modulation; however, dysbiosis of microbiota due to the tx and IS medicines is a contributing factor that decreases in the baseline predominant microbes and also results in a loss of overall diversity, and the emergence of few new dominant microbial populations (35). A very interesting finding from this study is that only 8 out of the 37 viruses identified in this dataset have been previously described in humans, and many of these have also been described to be pathogenic. Human infections have been described with some of the identified viruses, suggesting that their presence in urine may be relate to the pathogenesis of general systemic or the underlying renal injury. Junin virus (an arenavirus) infections can result in clinical human disease inclusive of fever, as well as an entity known as Argentine hemorrhagic fever (36). Tacaribe virus is an arenavirus that can also cause human fevers and hemorrhagic disease (36). The HHV-6 virus is a highly prevalent virus in children and causes fever, diarrhea, and rashes (37). Rhinoviruses are single-stranded RNA viruses that are the most infectious agent in humans and the most likely culprit for the common cold (38). *Acanthocystis turfacea* chlorella virus 1 has been in 44% of healthy humans (39) and has only been described in the oropharynx and is not known to be pathogenic to humans. The Marseille virus is a nucleocytoplasmic large DNA virus that has been described in blood and stool of patient with nonfebrile lymphadenopathy (40).

The SV40 virus is a DNA polyoma virus that was likely introduced into the human population through contaminated vaccines (41) and infection with this virus is frequently co-infected with BK virus infection, this being an almost invariant finding in the tx population. Recent studies have also highlighted the role of chronic SV40 infection with human cancers (42). Antibodies against the T antigen of SV40 cross reacts with the T antigens of BK and JC viruses, which are all in the polyoma virus family, and the SV40 stain is used for diagnosis of BK virus infection in BKVN. BK virus exposure, in the kidney, is seen in 90% of the normal population, and our data suggests that the urinary virome has traces of BK viral proteins in all the healthy controls sampled. BK virus replication is seen in 10–60% of kidney tx patients have been described to shed BK virus in their urine, which is confirmed by our data (Figure 2). BK virus is latent in renal tubular epithelial cells and its prevalence is known to increase in immune dysfunction and immunosuppression exposure (43). In this study, we could not identify any urinary virome specific differences that could distinguish the AR and BKVN groups, despite these conditions being immunologically distinct and requiring very different treatment approaches, i.e., minimization of immunosuppression in BKVN and increased immunosuppression in AR. The AR category was bx confirmed and there was negative SV40 stain in the bx tissue examined. Nevertheless, this data suggests that the detection of BKVN may be under-reported in kidney tx patients, and histological changes may be patchy resulting in BKVN disease underdiagnosis.

Of the remaining 19 viruses that have not been previously described in humans, the following have been noted to be used as pesticides/insecticides (*E. obliqua* nucleopolyhedrovirus, *Euproctis pseudoconspersa* nucleopolyhedrovirus, Helicoverpa armigera nucleopolyhedrovirus, *H. zea* single nucleopolyhedrovirus). Pesticides in public health use are intended to limit the potential for disease but they have been known to make their way into human tissues/bodily fluids if not properly handled and that organic diets limit the amount of pesticides in children urine (44, 45). The finding of these viral proteins in human urine samples raises a question of environmental exposure to these previously undiscovered, possible contaminants.

The finding of 100% prevalence of some viruses only in the healthy control group suggests that there are viral commensals that may exist either without harm or may even be hypothesized to assist in immune adaptation. Phylogentic tree analysis of these viruses may be interesting, as some viruses such as bacteriophages play an important role in health by removing pathogenic bacteria and helping boost innate immunity (16). A reduction in the protective viruses or phages may result in increased bacterial proliferation which is seen in the context of IS after kidney tx. Metagenomic sequencing of the new viral species found in tx patients under IS may identify if IS exposure has changed the resistance patterns or resistome of some of the viruses, especially those that are more prone to drive inflammation and immunity (16). Silver nanoparticle treatment has been shown to reduce gut viral and bacterial populations that are pro-inflammatory (46). Mucinophilic microbiomes may be more prone to provide non-host protective immunity, and the loss of any mucinophilic viruses/microbes with IS may also be a driver of the increased risk of cystitis and urinary tract infections seen in patients after tx.

The microbiome intersects with states of health and disease. The novel detection of a large number of viruses used as pesticides/insecticides is a surprising finding. Identification of viruses that are not expected to be present in the urine points to a possibility of their origin to enter bodily systems through ingestion (contamination of food, water, or other drinks such as milk) or cutaneous absorption (e.g., improper hand washing). In fact, according to federal law, a small residue of pesticides/insecticide contamination of human food is recognized and accepted (41, 47), even though no one to date has examined their presence in urine. It is also possible that these viruses colonize or invade the urinary tract by ascending infection, especially in the context of IS in a kidney tx patient, where there is lowered host immune defense.

Further validation of these results by evaluation of the human virome repertoire in different geographic and demographic cohorts of patients with different causes of kidney injury will better identify if the peculiar repertoire of viral proteins observed in this study is specific to a patient, their geography, their demography, or the kidney disease subtype. Additional validation studies by urine viral PCR assays can provide for rapid assessment of the clinical impact of these viruses in other kidney injury cohorts. Interestingly, in the repertoire of identified viral peptides, we did not observe any phages. This is attributed to the methodology used for peptide preparation from urine supernatant, which does not capture bacteria (which get pelleted out), the host of phage viruses (48). The virome report based on DNA sequence analysis also did not identify phages in the published report (19). The urine virome study that used purified bacteriophage isolation using cesium chloride density gradient has reported identification of phages (20). These articles all support that the methodology of sample prep influences the eventual findings.

Colonization, resistance, and microbial ecology has been described and well studied in the context of microbial infections (49), and it has been shown that antibiotic treatment (in the context of treating infections in the animals that form food products or as part of the antiviral prophylaxis for tx patients in the first 3–6 months post-tx) eliminates many commensal bacterial and viral species form the gut and other body fluids and reduces antimicrobial defense (50). Colonization by microbiota in early life shapes the immune system and create a window of opportunity for a homeostatic state, which if disrupted, in the context of tx and IS, can contribute to inflammation. Precision medicine approaches in the future may want to customize therapy from a “microbiota centered” and “host centered” approach to precision medicine. Until recently, we have not paid much attention to the food microbiome and the understanding that many emerging infections may be vectored by foods. Current efforts to develop a *metagenome trackr* tool that will evaluate the impact of different infections—bacterial, viral, and fungal and their fingerprints in the context of the food that we eat, that maybe influenced by ambient temperature, animal cohort poor survival causation, and agricultural practices inclusive of soil treatment (methyl bromide treatment of soil as a pesticide results in a huge increase in bacillus species), water sources [different microbial loads in pond vs. well water (51)], genetics of the food plant cultivar that may result in altered plant defense to microbes, plants, and pesticides (51). Viral and bacterial lipoproteins may be responsible for immune modulatory properties, and recent studies also suggest a role for the interaction of innate immunity for regulating microbiomal diversity and controlling infection, by specific cell lineages such as mucosal invariant T cells (MAIT cells). Hence, additional studies that focus on the causation of microbial diversity of human tissues and its impact on inflammation and immune responses will be paramount to conduct as part of future research.

## Ethics Statement

All the study samples were collected from pediatric and young adult recipients transplanted between years 2000 and 2011 at Lucile Packard Children’s Hospital of Stanford University. The study was approved by the ethics committees of Stanford University Medical School and UCSF Medical Center. All adult patients and parents/guardians of non-adult patients provided written informed consent to participate in the research, in full adherence to the Declaration of Helsinki.

## Author Contributions

MS and TS participated in the design of the study; SN, TS, NM, and MS participated in the writing of the article and the interpretation of the results; NM performed the statistical analyses and evaluation of results; CN, KB-J, and W-JQ generated and processed the raw data.

## Conflict of Interest Statement

The authors declare that the research was conducted in the absence of any commercial or financial relationships that could be construed as a potential conflict of interest. The reviewer DP declared a shared affiliation, with no collaboration, with three of the authors NM, TS, and MS to the handling editor.
